# Sustainable Additive Manufacturing of IN718 Blades: Powder Recycling Strategies for Reduced Carbon Footprint

**DOI:** 10.3390/ma18061344

**Published:** 2025-03-18

**Authors:** Xunchen Liu, Yilun Wang, Tengyuan Fang, Wenxuan Wang, Zhiheng Hu, Yang Meng, Bo Huang, Yuan Fang, Lin Hua, Mingzhang Chen

**Affiliations:** 1 School of Automotive Engineering, Wuhan University of Technology, Wuhan 430070, China; liuxunchen2021@163.com (X.L.);; 2 Hubei Longzhong Laboratory, Wuhan University of Technology, Xiangyang 441000, China; 3 School of Art and Design, Wuhan Institute of Technology, Wuhan 430070, China; 4 Key Laboratory of Catalysis and Energy Materials Chemistry of Ministry of Education, College of Chemistry and Materials Science, South-Central University for Nationalities, Wuhan 430074, China

**Keywords:** IN718 superalloy, powder recycling, additive manufacturing, life cycle assessment, sustainable manufacturing

## Abstract

With the growing demand for aero-engine turbine blades, the resource consumption and environmental impact of superalloy powder in the manufacturing process have become increasingly significant. This study focuses on IN718 nickel-based superalloy powder and establishes a recycling method based on powder mixing. By mixing sieved recycled powder with new powder at a 1:1 mass ratio, comprehensive characterization tests, including powder morphology analysis, particle size distribution, blade printability evaluation, mechanical property tests (tensile strength at both 25 °C and 650 °C), and microhardness measurements, demonstrated that the blended powder maintained performance characteristics comparable to new powder, with no statistically significant differences observed. Furthermore, this study introduces the life cycle assessment (LCA) methodology into the field of superalloy powder recycling, providing a novel technical approach for sustainable development in aerospace manufacturing. A quantitative analysis of environmental impacts throughout the blended powder recycling process indicates that this method can reduce carbon emissions by 45% and energy consumption by 48%.

## 1. Introduction

With the rapid development of the aerospace industry, turbine blades, as core components of aero-engines [1,2], have shown a continuously growing market demand [3,4]. As a critical raw material for turbine blade manufacturing [5,6], the demand for superalloy powder has increased significantly [7,8,9]. Specifically, with the widespread application of additive manufacturing technology in turbine blade production, the metal powder additive manufacturing market is projected to grow at a compound annual growth rate of 21.4% [10,11]. This has raised widespread concerns about resource utilization and environmental impacts during powder preparation processes [12,13].

In the superalloy powder preparation process [14,15], significant energy consumption is required throughout various stages, from raw material melting [16] to gas atomization [17], screening, and spheroidization treatments [18,19], accompanied by substantial environmental burdens [20]. With the advancing concept of sustainable development, achieving energy conservation, emission reduction, and environmental friendliness in powder production processes [21,22] while meeting rapidly growing market demands has become a critical research focus.

Recently, significant progress has been achieved in metal powder recycling technology across various material systems [23]. Denti et al. [24] demonstrated that Ti6Al4V powder maintains good sphericity and flowability after multiple recycling cycles, with the printed parts meeting aerospace requirements for mechanical properties. Research by R. Douglas [25] showed that 316L stainless steel powder exhibited only a slight increase in oxygen content after 15 recycling cycles, while the tensile strength and elongation of printed parts remained largely stable. Pereira et al. [26] further confirmed that recycled 316L powder could achieve mechanical properties and surface quality comparable to new powder through optimized process parameters. Studies on CoCrMo alloy powder recycling [27] indicated that recycled powder could maintain good biocompatibility and mechanical properties. Aluminum alloy powder retained over 95% of its mechanical properties and stable microstructure even after 20 recycling cycles [28]. However, research on nickel-based superalloy powder recycling remains limited.

Life cycle assessment (LCA), as a systematic environmental impact evaluation method, has been widely applied across various fields [29,30,31]. Currently, this methodology is well established in the new energy battery sector, as demonstrated by Wang et al. [32], who quantitatively analyzed carbon emissions throughout the life cycle of lithium-ion batteries. Feng et al. [33] assessed the environmental impacts of solid-state battery manufacturing processes. Liu et al. [34] verified the feasibility of bio-metallurgy through comparing different recycling processes. Sun et al. [35] developed an environmental benefit evaluation model for battery cascade utilization, while Xia et al. [36] investigated the life cycle impacts of sodium-ion batteries. However, in the field of metal powder additive manufacturing, particularly in superalloy powder recycling processes, there is a notable lack of life cycle assessment studies (including carbon footprint analyses). This paper introduces, for the first time, the LCA methodology to nickel-based superalloy powder recycling research. By establishing a comprehensive evaluation system, we quantitatively analyze the environmental impacts throughout the powder preparation, usage, and recycling processes, providing a new research paradigm and theoretical foundation for the sustainable development of superalloy powder.

Based on the above research background, this study establishes a recycling method for IN718 superalloy powder. This method creates a closed-loop powder recycling system by uniformly mixing screened recycled powder with new powder at a 1:1 mass ratio. It is important to note that this specific ratio was not chosen to optimize powder utilization efficiency or mechanical properties, but rather to investigate the fundamental feasibility of IN718 alloy powder recycling and its environmental implications. Our primary objective was to evaluate the practical viability of powder recycling and its potential benefits for carbon emission reduction and environmental protection. The research systematically examines key parameters including the morphology and size distribution of the mixed powder, as well as the formability, tensile strength, and microhardness of printed blades, comparing these with those of new powder to evaluate its feasibility in additive manufacturing processes. Through establishing a comprehensive life cycle assessment framework, we quantitatively analyze the improvements in environmental impact factors such as resource consumption and carbon emissions achieved by this recycling method. This mixed-powder recycling technology not only provides a new technical solution for sustainable utilization of superalloy powder but also offers important theoretical foundation and data support for sustainable development in the aerospace manufacturing sector.

## 2. Materials and Methods

### 2.1. Materials

**Figure 1:**
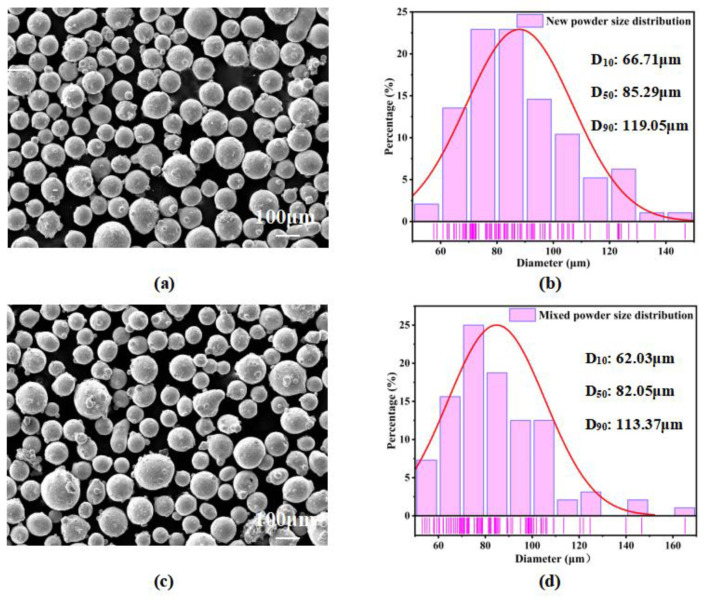
The morphology and particle size distribution of IN718 powders: (**a**) SEM image of new powder, (**b**) particle size distribution of new powder, (**c**) SEM image of mixed powder and (**d**) particle size distribution of mixed powder.

Gas-atomized IN718 metal powder supplied by AVIMETAL AM Technology (Beijing, China) Co., Ltd. was used as the experimental material. The chemical composition (wt.%) of the powder was provided by the supplier and independently verified using Inductively Coupled Plasma-Optical Emission Spectrometry (ICP-OES), with both sets of results showing close agreement, as detailed in Table 1. The morphological characteristics and particle size distribution of both virgin and mixed powders were analyzed using scanning electron microscopy (Figure 1a,c) and particle size analysis (Figure 1b,d). The brighter pink regions at the bottom of each image indicate areas of more concentrated particle size distribution. The particle size analysis revealed that the new powder primarily ranged from 66 to 119 µm in diameter (Figure 1b), while the mixed powder showed a size distribution of 62–113 µm (Figure 1d). A statistical analysis indicated that both powders exhibited similar morphological characteristics and particle size distributions.

**Figure 2:**
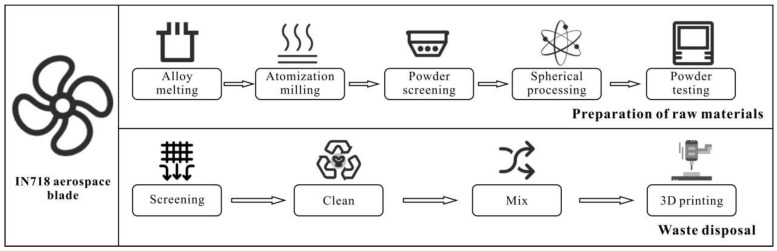
Schematic of the additive manufacturing process for IN718 aerospace blade production and powder recycling.

Figure 2 illustrates the detailed material preparation process flow of this study, which comprises two key stages: raw material preparation and powder recycling. The raw material preparation stage follows a sequential process including alloy melting, gas atomization, particle size screening, spheroidization treatment, and comprehensive powder characterization, ultimately producing IN718 initial powder material that meets additive manufacturing requirements. In the recycling stage, a closed-loop process system was established: residual and waste powder from the additive manufacturing process is collected, undergoes precise screening and surface cleaning treatments, and is then uniformly mixed with newly prepared powder at a 1:1 mass ratio before being reintroduced into the additive manufacturing process. This powder recycling strategy not only significantly improves material utilization efficiency but also achieves the optimization of manufacturing costs and a reduction in environmental burden, embodying the concept of sustainable manufacturing.

### 2.2. DED Strategy

**Figure 3:**
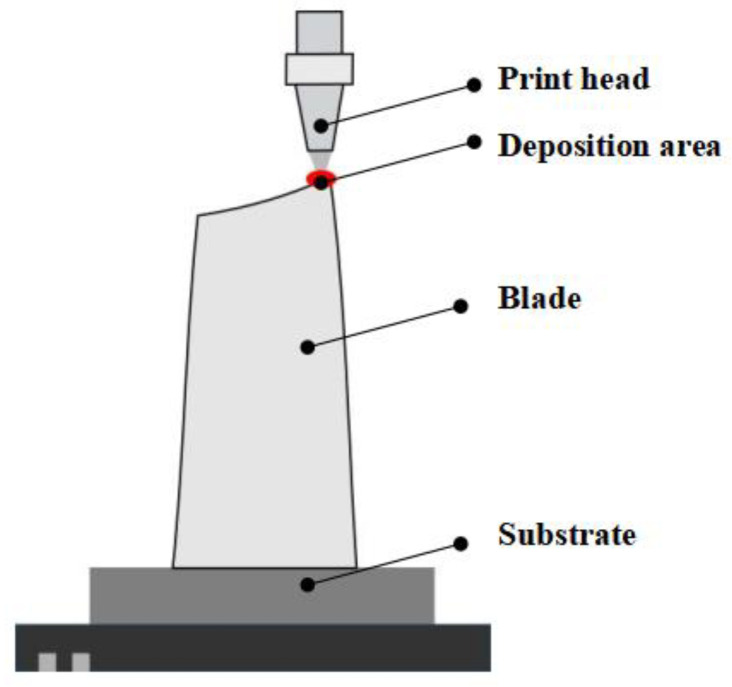
DED equipment working principle diagram.

As shown in Figure 3, this study employed directed energy deposition (DED) technology to fabricate turbine blade specimens. Through process parameter optimization, the optimal forming parameter combination was determined: laser power of 600 W, powder feed rate of 0.3 L/min, and scanning speed of 300 mm/min.

### 2.3. Numerical Simulation

This study aimed to conduct a comprehensive environmental impact assessment of IN718 powder recycling in additive manufacturing, with particular emphasis on evaluating the potential environmental benefits of implementing mixed powder strategies in aerospace component manufacturing. To achieve this objective, a typical aerospace component manufactured using the directed energy deposition (DED) process, weighing approximately 1.6 kg, was selected as the functional unit for analysis.

The system boundaries of this study encompass the entire manufacturing chain, from raw material extraction and processing to material transportation and manufacturing processes. Key manufacturing stages include powder atomization, screening, spheroidization treatment, DED printing, and powder recycling operations. The analysis primarily focuses on the production phase of the product lifecycle, as this phase has been identified as having the most significant environmental impact potential.

To ensure the reliability of the assessment, several fundamental assumptions were established. These include the maintenance of constant process parameters throughout the manufacturing cycle, transportation distances based on existing supply chain configurations, equipment operation at standard efficiency levels, stable environmental conditions during production, and consistency in material properties across different powder batches.

For the inventory analysis, the SimaPro 9.5 software program was employed in conjunction with the CML methodology for environmental impact assessment. The comprehensive inventory documentation includes material inputs (virgin powder consumption and recycled powder ratios), energy consumption (power requirements for atomization, printing, and auxiliary systems), process emissions (both direct and indirect), resource utilization (protective gases, carrier gases, and cooling systems), and waste streams (material losses and recyclable content).

## 3. Result

### 3.1. Printability of Blade Formation

**Figure 4:**
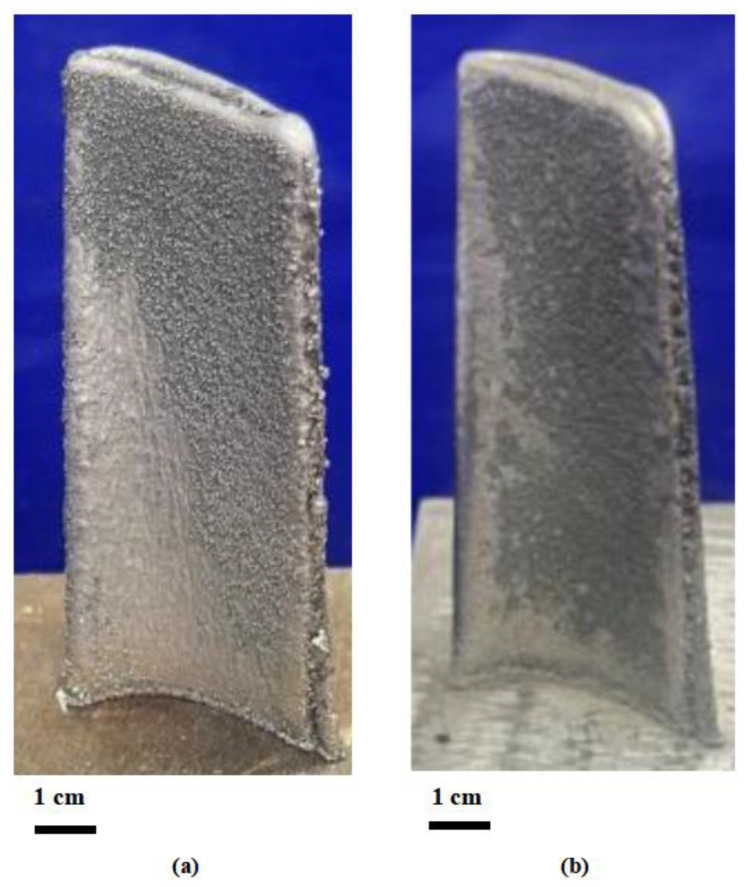
Macroscopic morphology of DED-printed aerospace blades: (**a**) using new powder, (**b**) using mixed powder.

Figure 4 presents high-magnification images comparing blade specimens fabricated using virgin powder (Figure 4a) and mixed powder (Figure 4b). The formability assessment was primarily based on the dimensional and geometric consistency between the two specimens, which showed minimal variations. These slight differences are considered acceptable for the manufacturing process, as the blade components undergo subsequent machining operations before final implementation.

### 3.2. Microhardness

**Figure 5:**
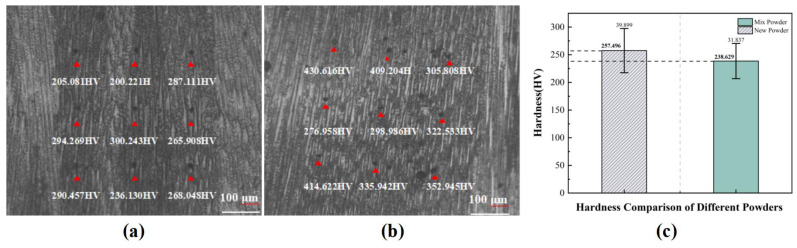
Macroscopic morphology of DED-printed aerospace blades: (**a**) using new powder, (**b**) using mixed powder and (**c**) comparison of average microhardness.

Microhardness testing was performed using a Huayin HV-1000A Vickers hardness tester (Laizhou Huayin Testing Instrument Co., Ltd., Laizhou, China). To ensure measurement representativeness and reliability, nine measurement points (red dots indicate indentation points) were systematically selected in both upper and lower regions of the samples, as shown in Figure 5a,b. A standardized measurement protocol was implemented: applying a 300 gf load with a dwell time of 15 s. The Vickers hardness value for each indentation point was obtained using the four-point measurement method to eliminate measurement errors and enhance data accuracy.

Figure 5c presents the microhardness distribution characteristics of turbine blades fabricated using virgin powder and mixed powder. The measurements revealed average microhardness values of 335.274 ± 35.298 HV for specimens produced with virgin powder and 321.963 ± 16.837 HV for those manufactured with mixed powder, indicating comparable mechanical properties between the two powder conditions.

The mechanical properties of components manufactured using mixed virgin and recycled IN718 powder were systematically compared with the published literature data to validate our recycling strategy. The hardness measurements of our mixed powder components (350–380 HV) demonstrated remarkable consistency with values reported in recent studies utilizing various manufacturing methods and powder conditions, as detailed in Table 2.

### 3.3. Tensile Properties

**Figure 6:**
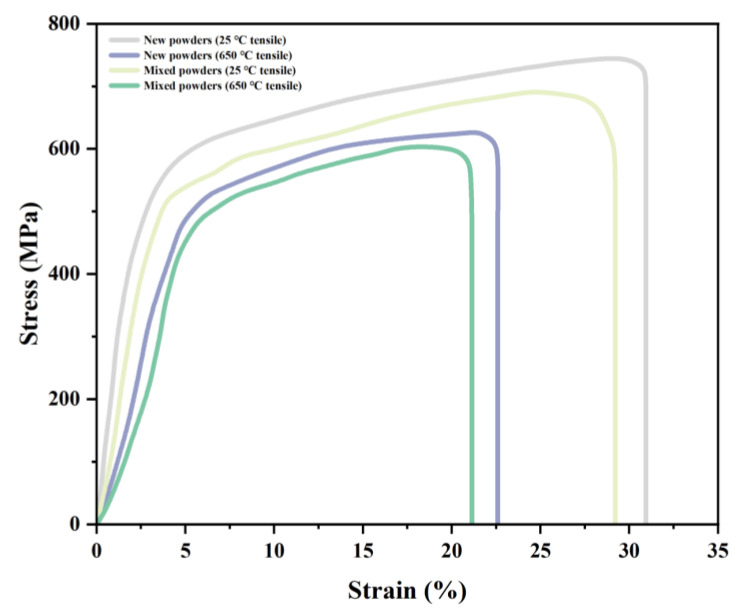
Tensile strength analysis of new and mixed powders at 25 °C and 650 °C.

Mechanical property testing was conducted using an electronic universal testing machine under both room temperature (25 °C) and elevated temperature (650 °C) conditions. Prior to high-temperature testing, specimens were held at 650 °C for 30 min to ensure a uniform temperature distribution throughout the sample. All tensile tests were performed under displacement-controlled mode with a loading rate of 0.5 mm/min using an electronic universal testing machine (Instron 3369, Instron Corporation, Norwood, MA, USA), while strain and load data were simultaneously collected using a high-precision extensometer (Epsilon 3542, Epsilon Technology Corp., Jackson, WY, USA) to generate comprehensive stress–strain curves. As shown in Figure 6, the comparative analysis of tensile properties revealed that specimens fabricated from mixed powder exhibited mechanical behavior closely matching that of new powder specimens under both testing conditions, with only minimal variations in performance characteristics.

**Figure 7:**
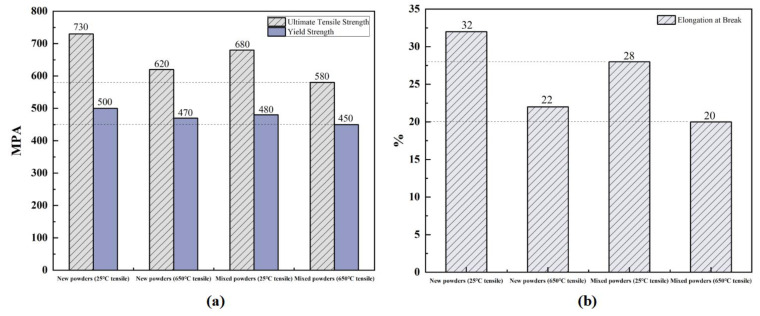
Tensile properties comparison of IN718 components at 25 °C and 650 °C: (**a**) elongation and (**b**) strength values.

As shown in Figure 7a, the mixed powder components exhibited mechanical properties comparable to those of virgin powder components. At room temperature, the ultimate tensile strength (UTS) of mixed powder components (680 MPa) was only slightly lower than that of virgin powder components (730 MPa), representing a minimal difference of approximately 6.8%. Similarly, the yield strength showed a marginal decrease from 500 MPa to 480 MPa, indicating excellent strength retention in the mixed powder components. At an elevated temperature (650 °C), both types of components maintained good mechanical properties. The UTS of mixed powder components (580 MPa) remained close to that of virgin powder components (620 MPa), with a difference of only 6.5%. The yield strength showed a similar trend, decreasing slightly from 470 MPa to 450 MPa. The elongation at break, as illustrated in Figure 7b, demonstrated good ductility retention in mixed powder components, with values of 28% at room temperature and 20% at 650 °C, compared to 32% and 22% for virgin powder components, respectively. These results indicate that mixing recycled powder with raw powder in a 1:1 ratio does not significantly impair the mechanical properties of manufactured components.

## 4. Discussion

The environmental impact assessment of aerospace component manufacturing has become increasingly critical for sustainable development. This study implements the life cycle assessment (LCA) methodology to evaluate the carbon footprint of nickel-based superalloy turbine blade manufacturing, where each component typically consumes 1.6 kg of metal powder material.

The system boundaries in this study encompass the direct manufacturing processes, including powder preparation, DED printing operations, post-processing procedures, and powder recycling systems. Key assumptions include steady-state operation conditions, consistent ambient environment, and uniform powder characteristics across batches. The system incorporates material flows (powder consumption and recycling), energy inputs (laser power and auxiliary systems), and immediate operational emissions. Elements outside the system boundaries include upstream processes (raw material extraction, powder production, transportation to facility) and downstream scenarios (component service life, end-of-life disposal) due to data availability constraints and focus on direct manufacturing impacts. The detailed simulation parameters are presented in the following Table 3.

**Figure 8:**
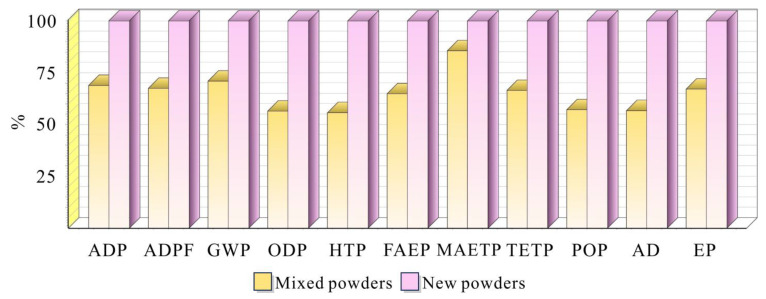
Characterization results analysis of new and mixed powders.

Figure 8 presents a comprehensive comparison of environmental impact characterization results between components manufactured using new and mixed powders. The analysis encompasses eleven key environmental impact categories: Abiotic Depletion Potential (ADP), Abiotic Depletion Potential for fossil fuels (ADPF), Global Warming Potential (GWP), Ozone Depletion Potential (ODP), Human Toxicity Potential (HTP), Freshwater Aquatic Ecotoxicity Potential (FAEP), Marine Aquatic Ecotoxicity Potential (MAETP), Terrestrial Ecotoxicity Potential (TETP), Photochemical Oxidation Potential (POP), Acidification Potential (AP), and Eutrophication Potential (EP).

The results demonstrate that components manufactured using mixed powders consistently exhibit lower environmental impacts across all categories, ranging from 50% to 85% of the impact values observed with new powders. This significant reduction is particularly notable in critical categories such as GWP, where mixed powder manufacturing shows approximately 30% lower impact compared to new powder processes. The consistent pattern across multiple environmental indicators suggests that the incorporation of recycled powder in the manufacturing process offers substantial environmental benefits while maintaining product quality, as previously demonstrated in mechanical property assessments.

**Figure 9:**
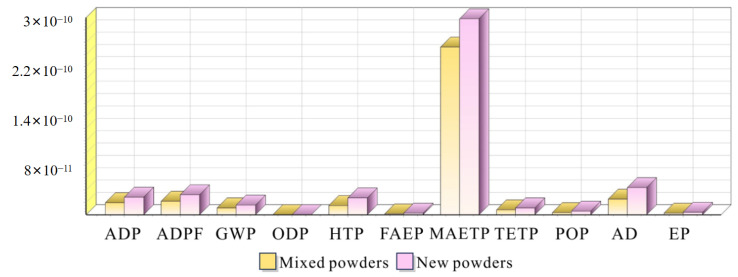
Normalization results analysis of new and mixed powders.

Figure 9 presents the normalized environmental impact analysis, complementing the characterization results shown in Figure 8. While Figure 8 demonstrates the consistent environmental advantages of mixed powder across all impact categories, the normalization analysis in Figure 9 further reveals the relative significance of each environmental impact category within the manufacturing system.

Both analyses conclusively demonstrate that the utilization of mixed powders leads to substantially reduced environmental impacts compared to new powders. The characterization results showed a 20–50% reduction across all environmental categories, and the normalization analysis confirms these benefits while identifying the most critical areas for environmental consideration. This comprehensive assessment validates the environmental advantages of incorporating recycled powder in the manufacturing process, supporting the development of more sustainable practices in aerospace component manufacturing without compromising product quality.

**Figure 10:**
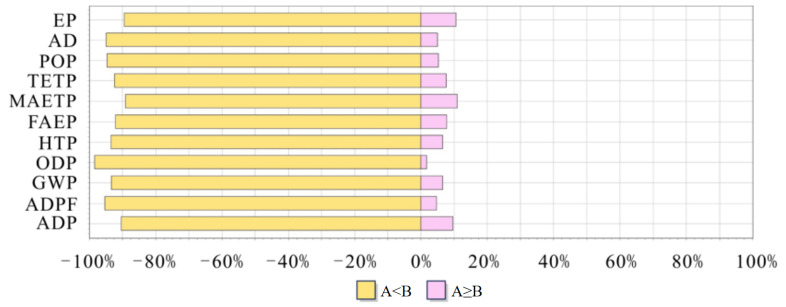
Uncertainty analysis of new and mixed powders.

Figure 10 presents an uncertainty analysis to validate the reliability of the environmental impact comparison between mixed (A) and new (B) powders. This statistical analysis evaluates the probability distribution of comparative results, where A < B indicates the likelihood that mixed powder shows lower environmental impact than new powder, while A ≥ B represents the opposite scenario. The uncertainty analysis demonstrates that across all environmental impact categories, there is a consistently high probability (approximately 80–90%) that mixed powder manufacturing yields lower environmental impacts compared to new powder processes.

Specifically, for critical indicators such as Global Warming Potential (GWP) and Marine Aquatic Ecotoxicity Potential (MAETP), the probability of mixed powder showing better environmental performance exceeds 85%. This robust statistical evidence reinforces our previous findings from both characterization and normalization analyses, providing strong confidence in the conclusion that mixed powder manufacturing represents a more environmentally sustainable approach. The consistently high probability of favorable outcomes across all environmental categories further validates the environmental benefits of incorporating recycled powder in the manufacturing process.

**Figure 11:**
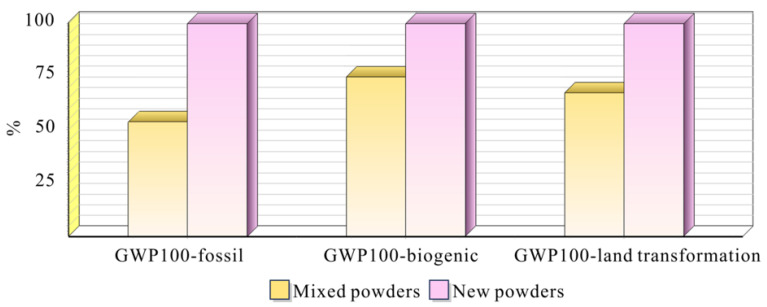
Carbon emission analysis of new and mixed powders.

Figure 11 provides a detailed analysis of carbon emissions across three distinct Global Warming Potential (GWP100) categories: fossil-based, biogenic, and land transformation emissions. The results demonstrate a consistent pattern where mixed powder manufacturing achieves significant carbon emission reductions compared to new powder processes. Most notably, the implementation of powder recycling technology leads to an overall 45% reduction in carbon emissions across all categories. The reduction is particularly evident in fossil-based emissions, where mixed powder manufacturing shows approximately 55% lower impact compared to new powder processes. Similar reduction patterns are observed in both biogenic and land transformation categories, with mixed powders consistently maintaining lower emission levels.

This substantial reduction in carbon emissions not only validates the environmental benefits of powder recycling but also demonstrates its practical significance in achieving sustainability goals in aerospace manufacturing. The comprehensive carbon emission analysis provides compelling evidence that the adoption of mixed powder technology represents an effective strategy for reducing the environmental footprint of aerospace component manufacturing while maintaining production efficiency.

## 5. Conclusions

This study demonstrated the feasibility of recycling IN718 nickel-based alloy powder in additive manufacturing through a systematic investigation. By implementing a 1:1 mixing ratio of recycled and new powders, comprehensive testing revealed that the blended powder maintained equivalent performance characteristics to virgin powder, including comparable powder morphology, size distribution, and mechanical properties of the manufactured components. Significantly, this recycling approach achieved a 45% reduction in carbon emissions and a 48% decrease in energy consumption, establishing an environmentally sustainable solution for IN718 powder reuse in additive manufacturing. Furthermore, this study establishes a recycling system for IN718 superalloy powder and introduces life cycle assessment methods, providing not only a novel technical solution for sustainable development in aerospace manufacturing but also crucial theoretical support and a research paradigm for green sustainable development in the aerospace industry. The main conclusions are as follows:

1. This study proposed and validated a powder recycling method through experimental verification, demonstrating highly similar morphology between both powder types and minimal differences in parameters such as the hardness and tensile properties of formed blades, meeting additive manufacturing process requirements.
2. We introduced the LCA methodology into the high-temperature alloy powder recycling field, establishing a corresponding environmental impact assessment system that systematically quantified 11 environmental indicators (including energy consumption, eutrophication, acidification, etc.).
3. The study systematically quantified the environmental benefits of mixed powder recycling, showing that this recycling method can achieve a 45% reduction in carbon emissions and a 48% decrease in energy consumption.
4. We provided data support for sustainable development in additive manufacturing, emphasizing the potential of mixed powder in improving environmental impact and offering theoretical support and research solutions for green sustainable development in the aerospace manufacturing sector.

## Figures and Tables

**Table 1:** Chemical composition of the as-received IN718 powder.

Element	C	Si	Cr	Co	Mo	Mn	Nb	Ti	Ta	Al	B	Ni
wt%	0.07	0.32	19.41	0.75	2.87	0.19	2.17	0.86	1.86	0.62	0.005	Bal.

**Table 2:** Hardness comparison of IN718 components using different powders and processes.

Reference	Manufacturing Method	Powder Type	Hardness (HV)
Nadiyadi et al. (2021) [37]	LPBF	Virgin	375–435
Chechik et al. (2022) [38]	SLM	Virgin	380–445
Kurdi et al. (2023) [39]	DED	Recycled	360–420
Marques et al. (2022) [40]	LPBF	Mixed	370–430
Ramiro et al. (2022) [41]	SLM	Virgin	385–450
Maurya et al. (2022) [42]	LPBF	Recycled	355–425
Tucho et al. (2021) [43]	DED	Mixed	350–415
Pramod et al. (2023) [44]	LPBF	Recycled	365–425

**Table 3:** System parameters and material-energy flow within DED manufacturing process boundaries.

Stage	I/O	Parameters	Quantity	Unit
Powder Preparation	Input	Virgin powder consumption	1	kg/component
	Input	Mixed powder ratio (virgin: recycled)	70:30:00	—
	Input	Sieving energy consumption	0.5	kWh/kg
	Input	Inert gas (Argon) consumption	2.5	L/min
	Input	Compressed air for cleaning	1.2	m^3^/h
DED Manufacturing	Input	Laser power	400	W
	Input	Scanning speed	600	mm/min
	Input	Powder feed rate	3.5	g/min
	Input	Carrier gas (Argon) flow rate	4	L/min
	Input	Shielding gas (Argon) flow rate	8	L/min
	Input	Cooling water circulation	2	L/min
	Input	Machine operation power	5.5	kWh/component
	Output	Metal component	0.85	kg/piece
	Output	Powder overspray	0.15	kg/piece
	Output	Process heat loss	15%	of input energy
	Output	Particulate emissions	0.02	g/component
Post-processing	Input	Surface finishing energy	1.2	kWh/component
	Input	Compressed air	0.8	m^3^/h
	Input	Cutting fluid	0.2	L/component
	Input	Tool wear	0.05	kg/component
	Input	Sieving energy	0.3	kWh/kg
	Input	Inert gas (Argon)	1.5	L/min
	Input	Collection system power	0.8	kWh/batch
	Output	Recovered powder	0.12	kg/component
	Output	Powder waste	0.03	kg/component
	Output	Filter dust	0.005	kg/component

## Data Availability

The authors attest that all data for this study are included in the paper.
